# Ipriflavone Suppresses Growth of Esophageal Squamous Cell Carcinoma Through Inhibiting mTOR *In Vitro* and *In Vivo*

**DOI:** 10.3389/fonc.2021.648809

**Published:** 2021-06-10

**Authors:** Xiaodan Shi, Yuanyuan Zhang, Xiaomeng Xie, Mengjun Pang, Kyle Laster, Jian Li, Xinli Ma, Kangdong Liu, Zigang Dong, Dong Joon Kim

**Affiliations:** ^1^ China-US (Henan) Hormel Cancer Institute, Zhengzhou, China; ^2^ Department of Pathophysiology, School of Basic Medical Sciences, Academy of Medical Science, College of Medicine, Zhengzhou University, Zhengzhou, China; ^3^ The Affiliated Cancer Hospital, Zhengzhou University, Zhengzhou, China; ^4^ The Collaborative Innovation Center of Henan Province for Cancer Chemoprevention, Zhengzhou, China; ^5^ International Joint Research Center of Cancer Chemoprevention, Zhengzhou, China

**Keywords:** patient-derived xenograft (PDX), ESCC, P70S6K, mTOR, Ipriflavone

## Abstract

Ipriflavone, a synthetic isoflavone that inhibits osteoclastic bone resorption, has been used clinically for the treatment of osteoporosis. However, the anticancer activity of Ipriflavone and its molecular mechanisms in the context of esophageal squamous cell carcinoma (ESCC) have not been investigated. In this study, we report that Ipriflavone is a novel mammalian target of rapamycin (mTOR) inhibitor that suppresses cell proliferation and induces cell apoptosis in ESCC cells. Ipriflavone inhibited anchorage-dependent and -independent growth of ESCC cells. Ipriflavone induced G1 phase cell cycle arrest and intrinsic cell apoptosis by activating caspase 3 and increasing the expression of cytochrome c. Based on the results of *in vitro* screening and cell-based assays, Ipriflavone inhibited mTOR signaling pathway through directly targeting mTOR. Knockdown of mTOR strongly inhibited the growth of ESCC cells, and the cell growth inhibitory effect exerted by Ipriflavone was found to be dependent upon mTOR signaling pathway. Remarkably, Ipriflavone strongly inhibited ESCC patient-derived xenograft tumor growth in an *in vivo* mouse model. Our findings suggest that Ipriflavone is an mTOR inhibitor that could be potentially useful for treating ESCC.

## Introduction

Esophageal carcinoma (EC) is one of the most aggressive gastrointestinal malignancies and the sixth leading cause of cancer-related deaths in men (1). Esophageal carcinoma is histologically classified into esophageal squamous cell carcinoma (ESCC), esophageal adenocarcinoma (EAC), and other subtypes, among which ESCC is the predominant histological subtype, accounting for nearly 90% of cases (2, 3). Despite significant advances in ESCC diagnosis and therapy, such as chemotherapy, surgical resection, and radiotherapy, the 5-year survival rate ranges from 15% to 25% (4). Although several types of therapies for the treatment of ESCC have been investigated, including molecular-targeting therapy and immunotherapy, the response rates of these therapies have been unsatisfactory due to the high risk of recurrence or late diagnosis (3, 5). Therefore, more effective treatment agents are desperately needed to enhance therapeutic response.

The mammalian target of rapamycin (mTOR)/V-Akt murine thymoma viral oncogene homolog (AKT) pathway is crucial for cell cycle progression, cell growth, survival, and metastasis in pathological and physiological conditions (6, 7). Abnormal activation of the mTOR/V-AKT pathway is related to numerous human malignancies (8). mTOR is a serine/threonine protein kinase that controls cell ribosomal biogenesis, cap-dependent translation, and protein synthesis through directly regulating p70S6 kinase (S6K), AKT, and elongation initiation factor 4E (eIF4E) binding protein-1 (4EBP1) (9–11). mTOR exists in two forms, TOR complex 1 and 2 (mTORC1 and mTORC2), respectively. mTORC1 and mTORC2 mutually share mTOR, mLST8/GβL (G-protein β-subunit-like protein), and DEPTOR (disheveled, egl-10, and pleckstrin domain-containing mTOR-interacting protein) subunits (12). However, mTORC1 contains RAPTOR (regulatory associated protein of mTOR) and PRAS40 (40 kDa Pro-rich Akt Substrate) subunits, while mTORC2 contains RICTOR (rapamycin-insensitive companion of mTOR), mSIN1 (mammalian stress-activated map kinase-interacting protein 1), and PROTOR (protein observed with RICTOR) subunits (13). mTORC2 is auto-phosphorylated at Serine 2481 which is located in a hydrophobic region near the conserved carboxyl-terminal FRAP tail (14, 15). Previous reports have indicated that phosphorylated mTOR is highly expressed and correlates with poor survival rate in ESCC patients (16). Therefore, mTOR is an important therapeutic target in ESCC and it is possible that mTOR inhibitors may be effective in treating ESCC.

Flavonoids are polyphenolic compounds that are extensively distributed in nature and found in a variety of fruits and vegetables (17). Flavonoids have been widely studied in the last few decades due to their potential anti-carcinogenic, anti-oxidant, and anti-inflammatory activities (18, 19). Ipriflavone, an isoflavone, has been shown to inhibit bone resorption and stimulate osteoblast activity (20, 21). It is clinically used for the treatment and prevention of osteoporosis in postmenopausal women (22). Recently, Ipriflavone analogs were reported to exhibit antitumor activity (23). Daidzein, the metabolite of Ipriflavone, exerts anti-tumor activity against bladder cancer cells *via* inhibition of the FGFR3 pathway (24). However, the inhibitory potential and associated underlying mechanisms of Ipriflavone in the context of ESCC have not been investigated. Here, we report that Ipriflavone is a potent mTOR inhibitor that suppresses ESCC growth *in vitro* and *in vivo*.

## Materials and Methods

### Cell Lines

KYSE30, KYSE70, KYSE450 and KYSE510 human esophageal squamous cell carcinoma (ESCC) cell lines were obtained from the Cell Bank of the Chinese Academy of Sciences (Shanghai, China). SHEE human normal esophageal cells were obtained from the Affiliated Cancer Hospital in Zhengzhou University. All cells were cytogenetically tested and authenticated before being expanded, aliquotted, and frozen. Each vial of frozen cells was thawed and maintained in culture for a maximum of 8 weeks. KYSE30 cells were cultured in a 1:1 mixture of RPMI1640 medium and Ham’s F12 medium with 2% fetal bovine serum (FBS) and 1% penicillin-streptomycin (PS). KYSE70, KYSE450 and KYSE510 were cultured in RPMI1640 medium with 10% FBS and 1% PS. The JB6 mouse epidermal cells were cultured in MEM/EBSS medium with 5% FBS and 1% PS. All cells were maintained at 37°C in a 5% CO_2_ humidified incubator.

### Reagents and Antibodies

Ipriflavone (7-isopropoxy-3-phenyl-4H-1-benzopyran-4-one) was purchased from TCI (Shanghai, China). AZD8055 was purchased from Selleckchem (Houston, TX, USA). Z-VAD-FMK was purchased from TOPSCIENCE (Shanghai, China). Aurintricarboxylic acid and BAX channel blocker were purchased from Santa Cruz Biotechnology (Santa Cruz, CA, USA). RPMI1640 medium and FBS were purchased from Biological Industries (Cromwell, CT, USA). Ham’s F12 medium was purchased from Lonza (Walkersville, MD, USA) and MEM/EBSS was purchased from Nanjing Keyjen Biotech (Nanjing, China). Active mTOR and p70S6K human recombinant proteins for kinase assays were purchased from SignalChem (Richmond, BC, Canada). Antibodies (1:1000) to detect phosphorylated AKT (S473), -mTOR (S2448), -mTOR (S2481), -EGFR (Y1068), -p70S6K (T389), -RSK(S380), -ERK1/2 (T202/Y204), total AKT, -mTOR, -EGFR, -p70S6K, -RSK, -ERK1/2, cyclin D3, p21, Cytochrome c, cleaved PARP and cleaved CASP3 were purchased from Cell Signaling Technology (Beverly, MA, USA). Antibodies to detect β‐actin (1:2000) were from Santa Cruz Biotechnology (Santa Cruz). Alanine aminotransferase Assay Kit and Aspartate aminotransferase Assay Kit was purchased from Nanjing Jiancheng Bioengineering Institute (Nanjing, China).

### Cell Proliferation

KYSE30 (1.5 × 10^3^ cells per well), KYSE450 (1.5 × 10^3^ cells per well), or KYSE510 (1.2 × 10^3^ cells per well) cells suspended in 100 μl complete growth medium (2% FBS for KYSE30 and 10% FBS for other cells) were seeded in 96‐well plates and incubated for 24 h. Cells were treated with various concentrations of Ipriflavone in 100 μl of complete growth medium. After incubation for 48 h, 20 μl of MTT solution (Solarbio, Beijing, China) were added to each well. After incubation for 2 hr at 37°C in a 5% CO_2_ incubator, the cell culture medium was removed. Subsequently, 150 μl of dimethyl sulfoxide (Merck Life Science, Shanghai, China) were added to each well, and formazan crystals were dissolved by gentle agitation. Absorbance was measured at 570 nm using the Thermo Multiskan plate‐reader (Thermo Fisher Scientific, Waltham, MA, USA).

### Foci Formation Assay

KYSE450 or KYSE510 cells were seeded in 6-well plates (800 cells per well) and incubated for 24 h at 37°C in a 5% CO_2_ incubator. Cells were treated with various concentrations of Ipriflavone for 10 days. Foci were fixed with 100% cold methanol and stained with 0.4% crystal violet (Solarbio) in methanol for 10 min at room temperature. Visible foci were tallied and analyzed.

### Anchorage‐Independent Cell Growth Assay

Cells (8 × 10^3^ cells per well) suspended in complete growth medium (RPMI1640) supplemented with 10% FBS and 1% gentamycin (Solarbio) were added to 0.3% agar with or without various concentrations of Ipriflavone in a top layer over a base layer of 0.6% agar containing the same concentration of Ipriflavone as the top layer. The cultures were maintained at 37°C in a 5% CO_2_ incubator for 2 weeks. Colonies were visualized using an inverted microscope and quantified using the Image‐Pro Plus software (v.6) program (Media Cybernetics,Rockville, MD, USA).

### Western Blotting

Cells were lysed in RIPA buffer (Solarbio) supplemented with 1mM PMSF (Solarbio) and quantified using the bicinchoninic acid assay (Solarbio). Proteins were separated by sodium dodecyl sulfate‐polyacrylamide gel electrophoresis and transferred to polyvinylidene difluoride membranes (Amersham Biosciences, Piscataway, NJ, USA). Membranes were blocked with 5% non-fat dry milk for 1 h at room temperature and incubated with the appropriate primary antibody overnight at 4°C. After washing with TBST, the membrane was incubated with a horseradish peroxidase‐conjugated secondary antibody at a 1:5000 dilution. Signals were detected with a chemiluminescence reagent (Amersham Biosciences) using the ImageQuant LAS4000 system (GE Healthcare, Piscataway, NJ, USA).

### Cell Cycle Assay

KYSE30, KYSE70 or KYSE510 (7 × 10^4^ cells per dish) cells were plated into 60‐mm culture dishes and incubated for 24 h. Cells were treated with different concentrations of Ipriflavone for 48 h in complete growth medium (2% FBS for KYSE30 or 10% FBS for KYSE70 and KYSE510 cells). Cells were collected by trypsinization and washed with phosphate‐buffered saline (PBS) and subsequently fixed in 1 ml of 70% cold ethanol. After rehydration, cells were incubated with RNase (100 μg/ml, Solarbio) and stained with propidium iodide (PI; 30 μM, Solarbio). PI staining was accomplished following the product instructions (Clontech, Palo Alto, CA) and the cells were analyzed by flow cytometry.

### Cell Apoptosis Assay

KYSE30 (4× 10^4^ cells per dish) and KYSE510 (5 × 10^4^ cells per dish) cells were seeded into 60‐mm culture dishes. After incubation for 24 h, cells were treated with different concentrations of Ipriflavone for 72 h. Cells were collected by trypsinization and washed with PBS. Cells were stained with Annexin V (BioLegnd, San Diego, CA) and PI. Apoptosis was analyzed by flow cytometry.

### *In Vitro* mTOR Kinase Activity

The active recombinant mTOR (50 ng) protein was mixed with different concentrations of Ipriflavone or control (DMSO) in reaction buffer (Cell Signaling Technology) and incubated at room temperature for 15 min. The inactive p70S6K recombinant protein (100 ng, Signalchem) and ATP (Cell Signaling Technology) were added and the mixtures were incubated at 30°C for 30 min. The reaction was stopped by adding 10 μl protein loading buffer and the mixture was separated by sodium dodecyl sulfate-polyacrylamide gel electrophoresis (SDS-PAGE, Solarbio). mTOR activity was detected by an p70S6K phosphorylation antibody.

### Computer Modeling

Molecular docking was performed using AutoDock Vina according to standard protocol (25). The receptor coordinates were taken from chain B of the mTORDeltaN-mLST8-ATPγS-Mg crystal structure (PDB 4JSP). Ligands and receptor PDB files were processed by the AutoDockTools-1.5.7 to generate the pdbqt files and to determine the grid space. Parameters were set to default values and ATP was used as a control in the docking analysis.

### Lentiviral Infection

Recombinant lentiviral viral vector (pLKO.1-mTOR) and packaging vectors (pMD2.0G and psPAX) purchased from Addgene Inc. (Cambridge, MA, USA) were transfected into HEK293T cells by using Lipofectamine 2000 (Invitrogen, Grand Island, NY, USA) according to the manufacturer’s protocol. KYSE450 and KYSE510 cells were infected with virus-containing media containing 21.3 μM of polybrene (Millipore, Billerica, MA, USA). After incubation for 24 h, the exhausted media was aspirated and cells were selected with complete growth media containing puromycin (1 μg/ml) for 48 h. The selected cells were used for experiments.

### ESCC Patient-Derived Xenograft Tumor Growth and Ethics Statement

Human ESCC tissues were obtained from the Affiliated Cancer Hospital in Zhengzhou University. The ESCC patients did not receive any chemotherapy or radiotherapy prior to surgery. Tissue histology was confirmed by a pathologist. Prior written informed consent was obtained from each patient. Severe combined immunodeficiency mice (SCID; Vital River Labs, Beijing, China) were maintained under “specific pathogen-free” conditions based on the guidelines established by Zhengzhou University Institutional Animal Care and Use Committee (Zhengzhou, China). To examine the effect of Ipriflavone on patient-derived ESCC tumor growth, ESCC tissues were cut into pieces (3–4 mm^3^) and implanted into the back of the neck of individual 6-9-week-old SCID mice. Mice were divided into 2 groups of 8 animals as follows: 1) vehicle (10% DMSO and 20% tween 80) group and 2) 100 mg Ipriflavine/kg body weight in vehicle. Ipriflavone or vehicle was administered by oral gavage once per day Monday through Friday. Tumor volume was calculated from measurements of the tumor base using the following formula: tumor volume (mm^3^) = (length ×width× height× 0.52). Mice were monitored until tumors reached 1.5cm^3^ total volume, at which time the mice were euthanized and tumors, liver, kidney, and spleen were extracted.

### Hematoxylin-Eosin Staining and Immunohistochemistry

The liver, spleen, kidney, and tumor tissues from mice were embedded in paraffin blocks and used for hematoxylin and eosin (H&E) staining or immunohistochemistry (IHC). For H&E staining, the tissue sections were deparaffinized, hydrated, and stained with H&E and then dehydrated. For IHC, tumor tissue sections were deparaffinized and hydrated. After antigen retrieval with 10 mM citrate acid and blocking with 5% BSA, the tumor tissue sections were hybridized with a primary antibody (Ki-67, 1:500, Thermo Fisher Scientific) overnight at 4°C and then a horse-radish peroxidase (HRP)-conjugated goat anti-rabbit or mouse IgG antibody (ZSGB-BIO, Beijing,China) was added and hybridized for 30 min. Tissue sections were developed with 3, 3′- diaminobenzidine (ZSGB-BIO) for 10 sec and then counterstained with hematoxylin for 1 min. Representative images of all sections were acquired by wide-field microscope and analyzed using the ImagePro Plus software (v. 6) program (Media Cybernetics).

### Statistical Analysis

All quantitative results are expressed as mean values ± SD or ± SE. Significant differences were determined using the Student’s t test or one‐way analysis of variance. A *p* value of <0.05 was considered to be statistically significant. The statistical package for social science (SPSS) for Windows (IBM, Inc. Armonk, NY, USA) was used to calculate the *p* value to determine statistical significance.

## Results

### Ipriflavone Inhibits ESCC Cell Growth

**Figure 1:**
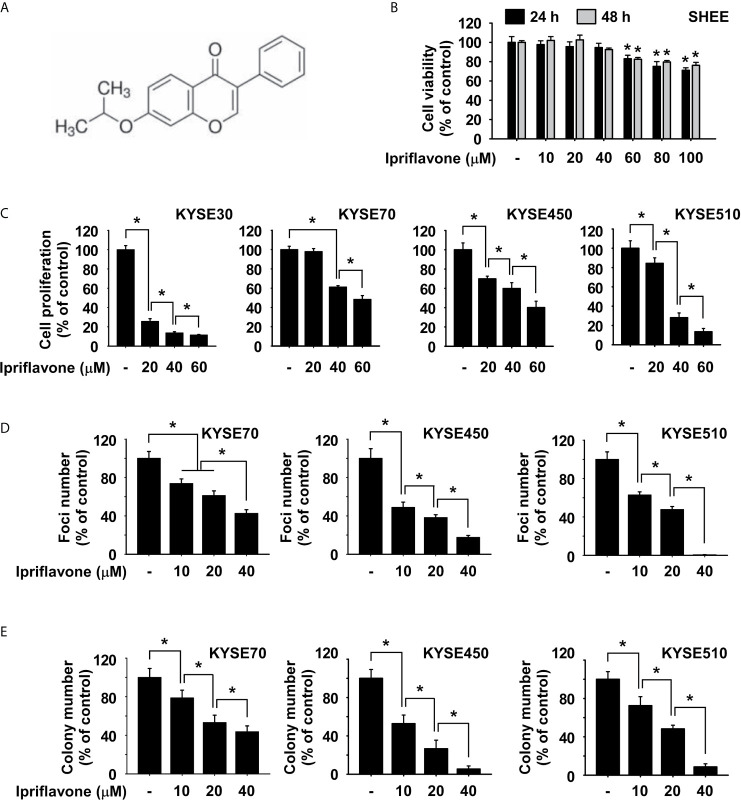
Ipriflavone inhibits ESCC cell growth. **(A)** Chemical structure of Ipriflavone. **(B)** Cytotoxicity of Ipriflavone on SHEE esophagus cells. Cells were treated with Ipriflavone at various concentrations for 24 h and 48 h. **(C)** Effect of Ipriflavone on ESCC cell growth. Cells were treated with Ipriflavone at various concentrations for 48 h. For **(B, C)**, cell growth was measured at an absorbance of 570 nm. **(D)** Effect of Ipriflavone on foci formation of ESCC cells. Cells were treated with Ipriflavone for 7 days and the number of foci was counted. **(E)** Effect of Ipriflavone on anchorage-independent growth of ESCC cells. Cells were treated with Ipriflavone and incubated for 2 weeks. Colonies were counted using a microscope and the Image-Pro PLUS (v.6) computer software program. For **(B, E)**, data are shown as means ± S.D. of triplicate values from 3 independent experiments and the asterisk (*) indicates a significant (*p* < 0.05) difference.

Ipriflavone is an isoflavone compound (**Figure 1A**). To determine the cytotoxicity of Ipriflavone, SHEE normal esophageal cells were treated with Ipriflavone at various concentrations and cell viability was analyzed by MTT assay. The results showed that SHEE cell viability was not obviously affected until cells were treated with Ipriflavone at concentration of 60 μM (**Figure 1B**). Next, we sought to determine the effect of Ipriflavone on anchorage-dependent cell growth and foci formation ability. KYSE30, KYSE70, KYSE450 or KYSE510 cells were treated with Ipriflavone at various concentrations and their growth was analyzed by MTT and foci formation assays. The results showed that the growth of ESCC cells was significantly decreased by Ipriflavone treatment in a dose-dependent manner (**Figures 1C, D**). We next assessed whether Ipriflavone could affect anchorage-independent ESCC cell growth using the soft agar assay. The results indicated that Ipriflavone strongly suppressed anchorage-independent cell growth of ESCC (**Figure 1E**).

### Ipriflavone Induces G1 Phase Cell Cycle Arrest and Reduces S Phase Cell Cycle in ESCC Cells

**Figure 2:**
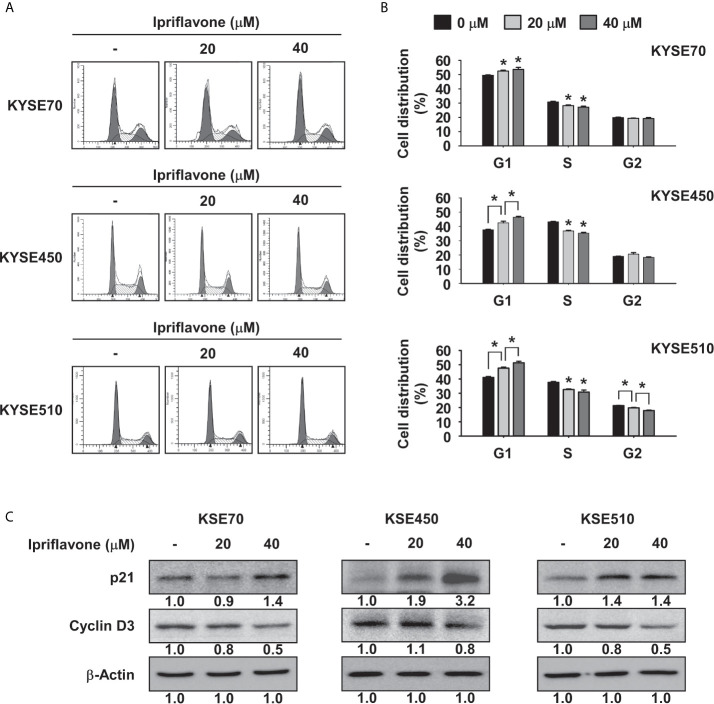
Ipriflavone induces G1 phase cell cycle arrest. **(A, B)** Effect of Ipriflavone on cell cycle in ESCC cells. KYSE70, KYSE450 and KYSE510 cells were treated with Ipriflavone for 48 h in 10% serum-supplemented medium. Cells were stained with propidium iodide (PI) and cell cycle was analyzed by Fluorescence Activated Cell Sorting (FACS). The bar graphs show the average DNA content corresponding to each cell cycle phase. For **(A, B)**, data are shown as means ± S.D. of triplicate values from 3 independent experiments and the asterisk (*) indicates a significant (*p* < 0.05) difference. **(C)** Effect of Ipriflavone on the expression of cell cycle marker proteins was determined by Western blotting. Band density was measured using the Image J (NIH) software program. For **(C)**, similar results were observed from three independent experiments.

To examine whether Ipriflavone could affect cell cycle regulation, ESCC cells were treated with Ipriflavone in complete growth medium for 48 h before being analyzed by flow cytometry. The results showed that Ipriflavone induced G1 phase cell cycle arrest and reduced the fraction of cells in S phase (**Figures 2A, B**). Furthermore, we also investigated whether the expression of cell cycle marker proteins was affected by Ipriflavone treatment. KYSE70, KYSE450 and KYSE510 cells were treated with different concentrations of Ipriflavone for 48 h and cell cycle marker proteins were analyzed after harvesting cells by Western blotting. The results indicated that Ipriflavone strongly increased the expression of p21, a marker protein of G1 phase, and reduced expression of cyclin D3, a marker protein of S phase (**Figure 2C**).

### Ipriflavone Induces Caspase-Mediated Apoptosis of ESCC Cells

**Figure 3:**
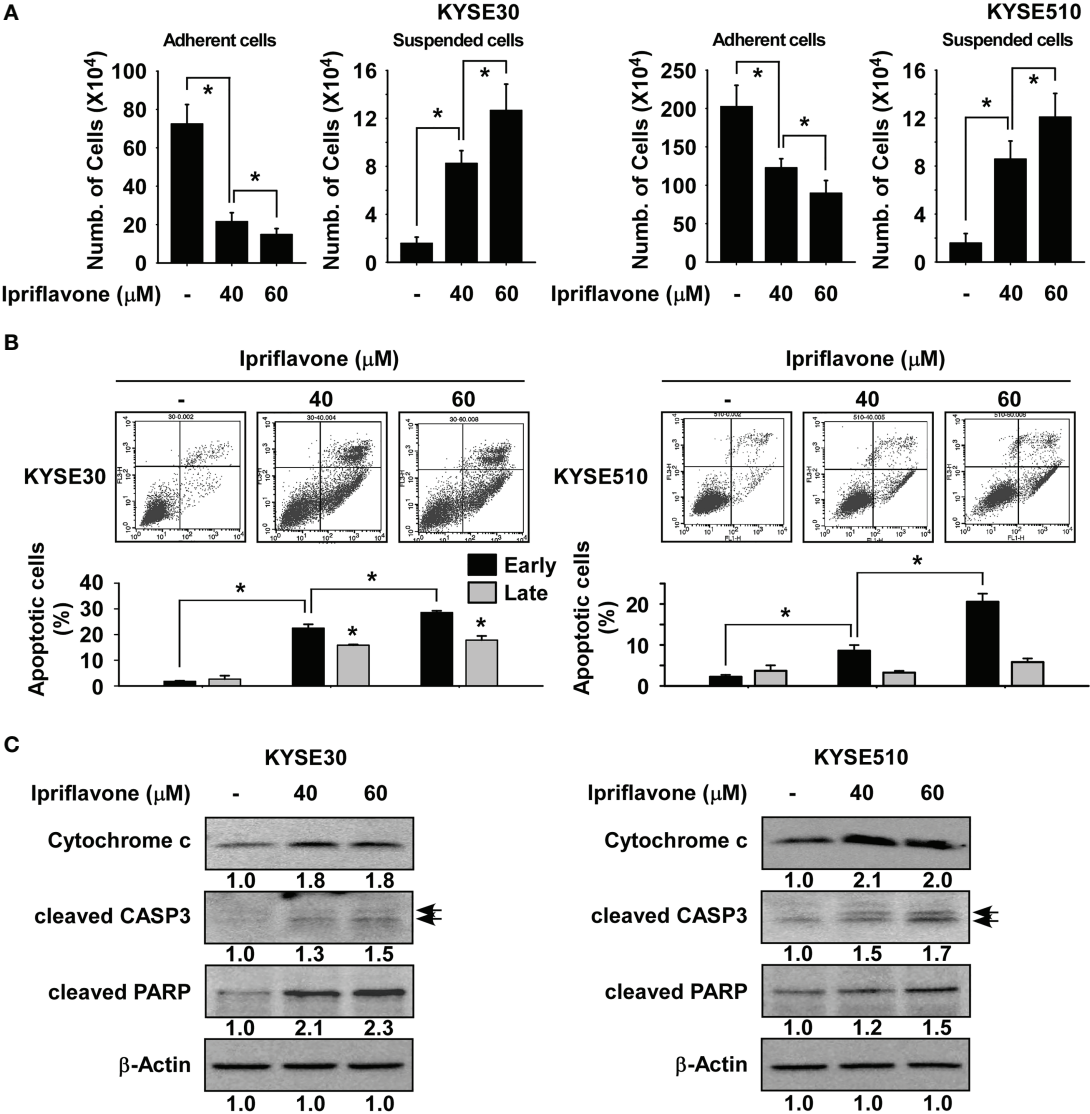
Ipriflavone induces apoptosis of ESCC cells. **(A)** Effect of Ipriflavone on cell death. Cells were treated with Ipriflavone for 72 h in 10% serum-supplemented medium. The number of suspended or attached cells was determined using a hematocytometer. **(B)** Effect of Ipriflavone on cell apoptosis. Cells were treated with Ipriflavone for 72 h in 10% serum-supplemented medium. Cells were stained with Annexin V and PI and then apoptosis was determined by FACS. For **(A, B)** data are shown as means ± S.D. of triplicate values from 3 independent experiments and the asterisk (*) indicates a significant (*p* < 0.05) difference. **(C)** Effect of Ipriflavone on apoptosis marker proteins. The expression of cytochrome c, cleaved CASP3 and cleaved PARP was determined by Western blotting. Similar results were obtained from three independent experiments and band density was measured using the Image J (NIH) software program.

To investigate the effect of Ipriflavone on ESCC cell death, suspended and adherent ESCC cell fractions were quantified after treatment with Ipriflavone treatment for 72 h. The results showed that the number of suspended cells was significantly increased in Ipriflavone-treated cells compared with control cells and that cell adherence was strongly decreased in a dose-dependent manner (**Figure 3A**). To determine whether Ipriflavone-induced ESCC cell death was due to apoptosis, the expression of annexin V was analyzed by flow cytometry in cells treated with Ipriflavone for 72 h. Results showed that early apoptosis in Ipriflavone-treated cells were significantly increased compared to control cells (**Figure 3B**). We next examined whether Ipriflavone could induce caspase-mediated cell death and apoptosis. The results indicated that Ipriflavone combined with Z-VAD-FMK (caspase inhibitor)-treated cells were resistant to the effect of Ipriflavone on cell death and early cell apoptosis compared to Ipriflavone-treated cells (**Supplemental Figures 1A, B**). However, Ipriflavone combined with aurintricarboxylic acid (topoisomerase II inhibitor) or BAX channel blocker-treated cells were sensitive to the effect of Ipriflavone on early cell apoptosis compared to Ipriflavone-treated cells (**Supplemental Figure 2**). We next determined the effect of Ipriflavone on the expression of apoptotic signaling molecules by Western blotting. Results showed that the expression of cleaved caspase 3, cleaved PARP, and cytochrome c was strongly induced by Ipriflavone treatment (**Figure 3C**).

### Ipriflavone Is a Potential mTOR Inhibitor

**Figure 4:**
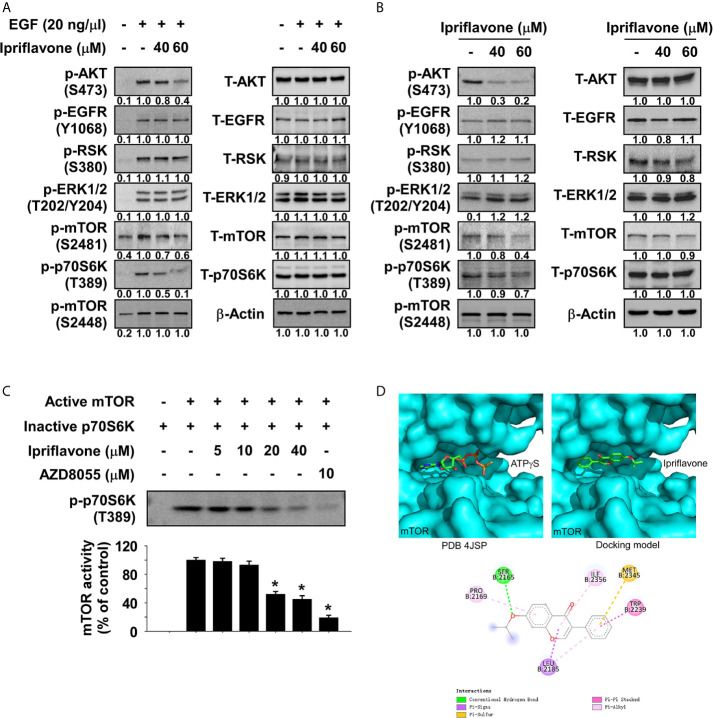
Ipriflavone is a novel mTOR inhibitor. **(A)** Effect of Ipriflavone on EGF‐induced kinase signaling molecules in JB6 cells. After serum starvation for 24 h, cells were treated with different doses of Ipriflavone for 6 h followed by EGF treatment for 30 min. various signaling molecules were analyzed by Western blotting. **(B)** Effect of Ipriflavone on various signaling molecules in KYSE450 ESCC cells. Cells were treated with Ipriflavone for 24 h and signaling molecule proteins were examined by Western blotting. **(C)** Effect of Ipriflavone on mTOR kinase activity was assessed by an *in vitro* kinase assay using active mTOR and inactive p70S6K proteins. The activity of mTOR was determined by Western blotting using a phosphorylated p70S6K antibody. AZD8055 (mTOR inhibitor) was used as a positive control. For all data, similar results were observed from three independent experiments and band density was measured using the Image J (NIH) software program. The asterisk (*) indicates a significant (*p* < 0.05) difference. **(D)** Modeling of Ipriflavone binding with mTOR. ATPγS (**D**, *upper left panel*) and Ipriflavone (**D**, *upper right panel*) binding with mTOR at the ATP binding pocket. (**D**, *lower panel*) Ligand Interaction Diagram (LID) of the binding. The Ipriflavone is shown as stick. LID legend is shown below.

To investigate the potential target proteins of Ipriflavone, a 28 panel *in vitro* kinase assay (Kinase profiling service, eurofins, https://www.eurofins.com) was performed using recombinant active kinase proteins with the specific substrates for each kinase in the presence or absence of Ipriflavone. Results indicated that Ipriflavone suppressed the activity of mTOR protein by nearly 40%, whereas the activity of other kinases were not significantly affected (**Supplemental Figure 3**). To examine the effect of Ipriflavone on the mTOR signaling pathway, JB6 cells were serum -starved for 24 h and subsequently treated with Ipriflavone for 6 h prior to treatment with EGF for 30 min. Results showed that EGF induced phosphorylation of AKT, while mTOR at S2481 and p70S6K were strongly inhibited in Ipriflavone-treated cells; other signaling molecules, especially phosphorylated mTOR at S2448, were not significantly affected (**Figure 4A**). To determine whether Ipriflavone could affect mTOR signaling pathways, KYSE450 ESCC cells were treated with Ipriflavone for 36 h before the expression of mTOR signaling proteins were analyzed by Western blotting. Results showed that Ipriflavone strongly suppressed the phosphorylation of mTOR (S2481), AKT, and p70S6K in a dose‐dependent manner, whereas other signaling proteins were not significantly affected (**Figure 4B**). The phosphorylation of p70S6K or AKT are directly regulated by mTORC1 or mTORC2 activity. Therefore, to confirm the effect of Ipriflavone on mTOR activity, we performed an *in vitro* kinase assay using a recombinant active mTOR protein and an inactive p70S6K protein. The results indicated that Ipriflavone significantly suppressed the phosphorylation of p70S6K by directly targeting mTOR (**Figure 4C**). To better understand the mechanism of mTOR kinase inhibition by Ipriflavone, molecular docking was performed using AutoDock Vina. Based on the docking model, Ipriflavone occupies the mTOR ATP binding pocket similar to the ATPγS in the co-crystal structure (PDB 4JSP) (**Figure 4D**, upper panel). The Ipriflavone top binding pose gave a predicted binding affinity (-7.4 kcal/mol) similar to that of ATP (-6.9 kcal/mol), suggesting Ipriflavone might be a ligand no worse than the ATP. The Ipriflavone-mTOR interactions are mediated by both hydrogen bond and hydrophobic interactions (**Figure 4D**, lower panel).

### The Inhibition of ESCC Cell Growth by Ipriflavone Is Dependent on the mTOR Signaling Pathway

**Figure 5:**
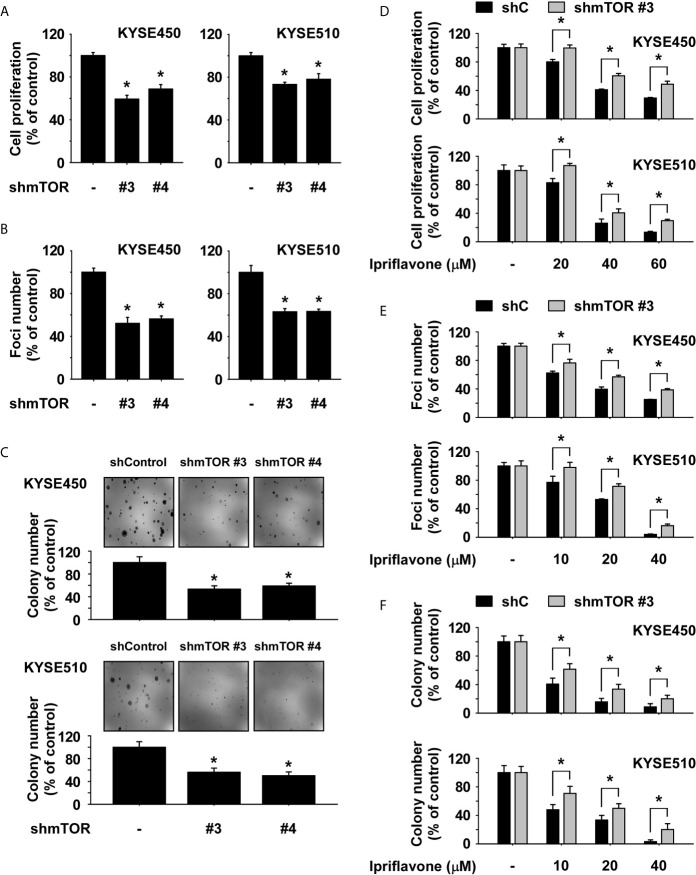
The inhibition of ESCC cell growth by Ipriflavone is dependent on the mTOR signaling pathway. Effect of mTOR knockdown on anchorage-dependent growth **(A)**, number of foci formation **(B)** and anchorage-independent growth **(C)** in ESCC cells. Cells were seeded and incubated for 48 h, 7 days or 2 weeks respectively **(A–C)**. Effect of Ipriflavone on ESCC cell growth was assessed in KYSE450 and KYSE510 stable cell lines that express shmTOR #3 or shControl. Cells were treated with Ipriflavone for 48 h **(D)**, 7 days **(E)** or 2 weeks **(F)** respectively, and cell growth was determined by **(D)** MTT assay, **(E)** foci formation or **(F)** soft agar assay. All data are shown as means ± S.D. of triplicate values from 3 independent experiments and the asterisk (*) indicates a significant (*p* < 0.05) difference.

To determine the levels of mTOR and p70S6K protein expression, protein lysates derived from SHEE normal esophageal and ESCC cells were analyzed by Western blotting. The results showed that phosphorylated mTOR was highly expressed in ESCC cells compared to SHEE cells (**Supplemental Figure 4A**). Next, to assess the effect of mTOR knockdown on ESCC cell growth, we established stable cell lines expressing either a control shRNA or an shRNA targeting mTOR in KYSE450 and KYSE510 cells and determined the expression level of mTOR protein by Western blotting. The results showed that expression of phosphorylated and total mTOR was strongly reduced in mTOR knockdown (KD) cells (**Supplemental Figure 4B**). To determine whether ESCC cell growth could be affected by knockdown of mTOR, anchorage-dependent and -independent growth of mTOR KD ESCC cells was evaluated by MTT (**Figure 5A**), foci-formation (**Figure 5B**) and soft agar assays (**Figure 5C**). The results showed that ESCC cell growth was significantly inhibited in mTOR KD cells compared to shControl cells (**Figures 5A–C**). Additionally, to examine whether Ipriflavone could inhibit mTOR signaling pathway-dependent ESCC cell growth and early cell apoptosis, ESCC cells expressing shmTOR #3 or shControl were treated with Ipriflavone for 48h (MTT assay; **Figure 5D**), 7 days (Foci formation; **Figure 5E**), 2 weeks (Soft agar assay; **Figure 5F**) or 72 h (**Supplemental Figure 5**). The results indicated that ESCC cells expressing shmTOR#3 were resistant to the inhibitory effect of Ipriflavone on cell growth and early cell apoptosis compared to cells expressing shControl (**Figures 5D–F** and **Supplemental Figure 5**).

### Ipriflavone Inhibits ESCC Patient-Derived Xenograft Tumor Growth *In Vivo*

**Figure 6:**
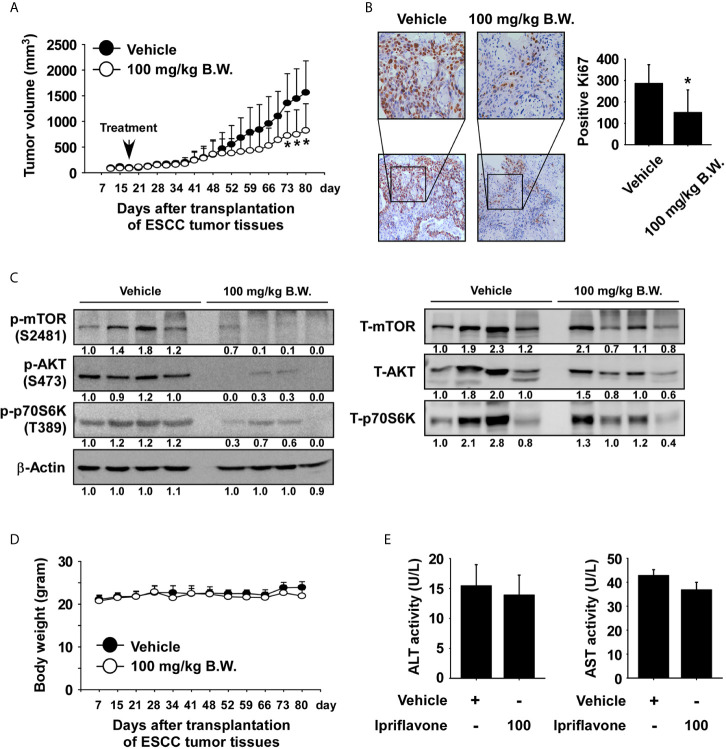
Ipriflavone inhibits ESCC patient‐derived tumor growth *in vivo*. Mice were divided into two groups for assessing the effect of Ipriflavone on ESCC PDX tumor growth. Groups are as follows: 1) vehicle group or 2) group treated with 100 mg/kg of Ipriflavone. Tumor-bearing mice were orally administered (by gavage) Ipriflavone or vehicle once a day Monday through Friday for 62 days. Tumor volumes were measured on the days indicated. **(A)** The effect of Ipriflavone on ESCC tumor growth. **(B)** Effect of Ipriflavone on Ki‐67 expression. Vehicle and Ipriflavone groups of tumor tissues were stained with Ki‐67 antibody (×40, ×100 magnification, *left panel*). The number of Ki‐67‐stained cells was counted from immunohistochemistry results (n = 6; * P < 0.05) (*right panel*). **(C)** Effect of Ipriflavone on the mTOR signaling pathway. Vehicle and Ipriflavone groups of tumor tissues were analyzed by Western blotting. **(D)** Effect of Ipriflavone on mouse body weight. Body weights of mice were obtained once a week. Data are shown as means ± S.E. of values obtained from the experiments. **(E)** Effect of Ipriflavone on ALT and AST activity. Before sacrifice mice, blood from vehicle and Ipriflavone groups were collected and analyzed. All data are shown as means ± S.E. of values obtained from the experiment groups. The asterisk (*) indicates a significant difference between tumors from vehicle-treated group or Ipriflavone-treated group mice as determined by t test (*p* < 0.05).

We next investigated whether Ipriflavone could inhibit ESCC patient-derived xenograft tumor growth *in vivo*. Human ESCC tumor tissues were implanted into the back of the neck of SCID mice. Mice were orally administrated Ipriflavone or vehicle 5 times per week over a period of 62 days. The results showed that administration of Ipriflavone significantly inhibited the growth of ESCC tumors relative to the vehicle-treated group (**Figure 6A**; *p* < 0.05). We next investigated whether Ipriflavone affects the expression of Ki-67 as a tumor proliferation marker protein by using immunohistochemistry. Results indicated that the expression of Ki-67 was significantly decreased in the Ipriflavone-treated group compared with the vehicle-treated group (**Figure 6B**). To determine whether the inhibitory effect of Ipriflavone treatment on the mTOR signaling cascade observed *in vitro* could be recapitulated *in vivo*, PDX tumor tissues were analyzed by Western blotting. Results confirmed that the protein levels of phosphorylated mTOR, AKT, and p70S6K were strongly reduced in the Ipriflavone-treated group compared with the vehicle-treated group (**Figure 6C**). Next, to determine the potential toxicity of Ipriflavone, the body weight of Ipriflavone-treated or untreated mice was measured once a week. The result showed that Ipriflavone-treated mice had no significant loss of body weight compared with the vehicle-treated group (**Figure 6D**). Additionally, the liver, spleen and kidney tissue were stained with hematoxylin and eosin (H&E) to further confirm whether Ipriflavone exhibits toxicity in SCID mice. Results indicated no distinct morphological changes in the Ipriflavone-treated group compared to vehicle-treated group mice (**Supplemental Figures 6A–C**). Furthermore, to determine effect of Ipriflavone on the liver toxicity, the activity of alanine transaminase (ALT) and aspartate transaminase (AST) liver enzymes were analyzed. Ipriflavone had little effect on the activity of ALT and AST (**Figure 6E**).

## Discussion

In the present study, we assessed the effects of Ipriflavone on ESCC cell growth and ESCC PDX tumor growth. We report that Ipriflavone, a clinical drug, may have therapeutic properties in ESCC patients, and may produce fewer serious adverse effects in patients compared with other mTOR inhibitors. The findings might provide significative insights and supporting evidence for ESCC chemoprevention and chemotherapy.

mTOR is constitutively activated in 25% of ESCC patients (16), and therapeutic targeting of the mTOR signaling cascade has increased survival rates and improved quality of life in ESCC patients (26). Additionally, an mTOR inhibitor strongly reduced ESCC cell growth, and induced G1 phase cell cycle arrest and cell apoptosis (**Supplemental Figures 7A–E**). Previously, rapamycin analogs (Temsirolimus and Everolimus) have been shown to exhibit anti-cancer activity through binding to FKBP12, thereby inhibiting mTORC1 (27). However, inhibition of mTORC1 may lead to feedback activation of IGFR and AKT (28). Interestingly, our findings suggested that Ipriflavone treatment did not induce feedback activation of AKT activation (**Figures 4A, B**). In addition, dual PI3K/mTOR inhibitors have been developed to overcome the paradoxical increase of PI3K induced by mTOR inhibitors, such as NVP-BEZ235 (29). However, these drugs also face the challenge of drug resistance after long-term treatment. The mTOR inhibitors AZD8055 and NVP-BEZ235 could be evacuated by ATP-binding cassette (ABC) transporters that decrease the intracellular levels of drugs, thereby contributing to poor treatment outcomes (30). Additionally, everolimus or AZD8055 can increase EGFR activation in tumor cells, leading to drug resistance (31). However, Ipriflavone did not affect EGFR activation (**Figures 4A, B**). Therefore, we suggest that Ipriflavone as a clinical mTOR inhibitor may not lead to drug resistance. We will investigate whether Ipriflavone could affect drug- resistant signaling pathway and expression of drug-resistant transporter genes after long-term treatment.

The results of our *in vitro* kinase assay and cell-based assays indicated that Ipriflavone directly suppressed the activity of mTOR (**Figure 4C**) and reduced expression level of mTOR at the S2481 residue (autophosphorylation site), AKT at the S473 residue (activation by mTORC2), and p70S6K at the T389 residue (activation by mTORC1) (**Figures 4A, B**). However, the expression level of mTOR phosphorylation at the S2448 residue (activation by AKT) did not change significantly (**Figures 4A, B**). Additionally, the results of the 28 *in vitro* kinase panel showed that Ipriflavone could not affect PI3K/PDK1 activity (**Supplemental Figure 3**). Therefore, our findings suggest that Ipriflavone directly inhibited mTORC1 and mTORC2, but not the PI3K/PDK1 signaling cascade. We will further investigate expression of mTORC1 and mTORC2-dependnet target genes, and also other molecular targets of Ipriflavone.

Apoptosis can be induced by intrinsic or extrinsic signaling pathways (32, 33). The release of cytochrome c, cleaved caspase 3, and PARP play a central role in the intrinsic cell apoptosis pathway (34). Knockdown of mTOR effectively induces cell apoptosis in ESCC cells (35). Inhibition of mTORC1 by small molecules induces intrinsic apoptosis through regulating translation of mitochondrial fission process 1 (MTFP1) (36). Therefore, we investigated whether Ipriflavone could induce intrinsic cell apoptosis. Results suggested that Ipriflavone strongly increased intrinsic cell apoptosis through inducing cytochrome c release and activating cleaved caspase 3 and cleaved PARP (**Figures 3B, C**).

The patient-derived xenograft (PDX) model has been reported to enhance predictability of tumor response to anticancer agents (37, 38). We first investigated the anti-tumor effect of Ipriflavone on ESCC PDX growth. The results showed that Ipriflavone significantly reduced ESCC tumor growth (**Figure 6A**). These findings suggest that Ipriflavone is a reagent that may be useful in clinically treating ESCC.

## Data Availability Statement

The raw data supporting the conclusions of this article will be made available by the authors, without undue reservation.

## Ethics Statement

The animal study was reviewed and approved by Zhengzhou University Institutional Animal Care and Use Committee (Zhengzhou, China).

## Author Contributions

XS and YZ performed the *in vitro* experiments and assisted with the cell based and *in vivo* experiments and prepared the manuscript. XX and MP assisted with the cell-based assays and the *in vivo* experiments. JL and XM performed the computer modeling. KDL and KLa performed the data analysis and manuscript editing. ZD and DJK supervised the overall experimental design and provided the idea. All authors contributed to the article and approved the submitted version.

## Funding

This work was supported by Henan Joint Fund, National Natural Science Foundation China (NSFC) [grant number U1804196 and 82073075] and Youth Science Foundation of Natural Science Foundation of Henan Province, China [grant number 212300410315].

## Conflict of Interest

The authors declare that the research was conducted in the absence of any commercial or financial relationships that could be construed as a potential conflict of interest.

The reviewer LL declared a shared affiliation with the authors to the handling editor at the time of review.
