# Supplementary figures and images for: Ipriflavone Suppresses Growth of Esophageal Squamous Cell Carcinoma Through Inhibiting mTOR In Vitro and In Vivo
Source: Front Oncol. 2021 Jun 10;11:648809. doi: 10.3389/fonc.2021.648809 (PMC8222593; doi:10.3389/fonc.2021.648809)

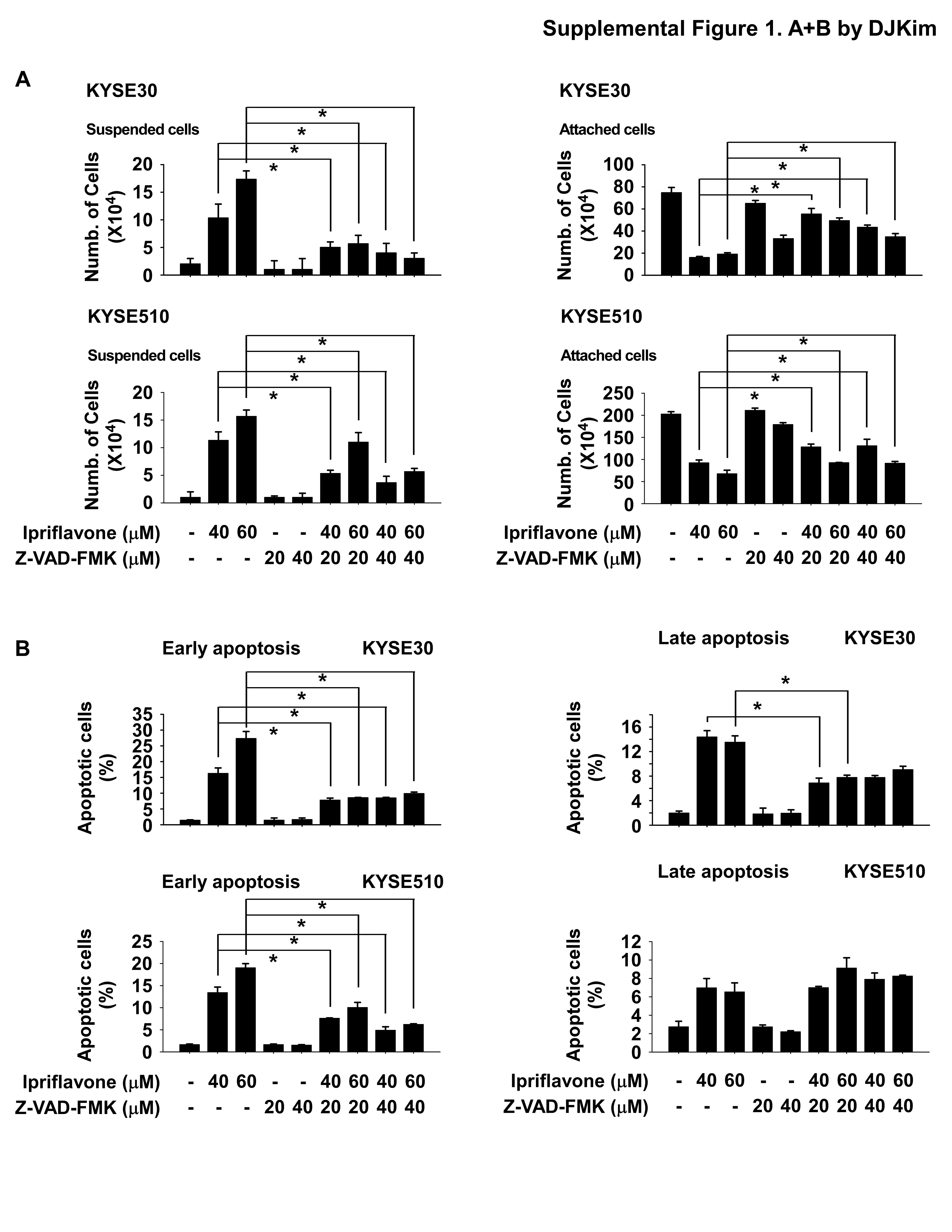

Supplement: Supplementary file 1 [file Image_1.jpeg]

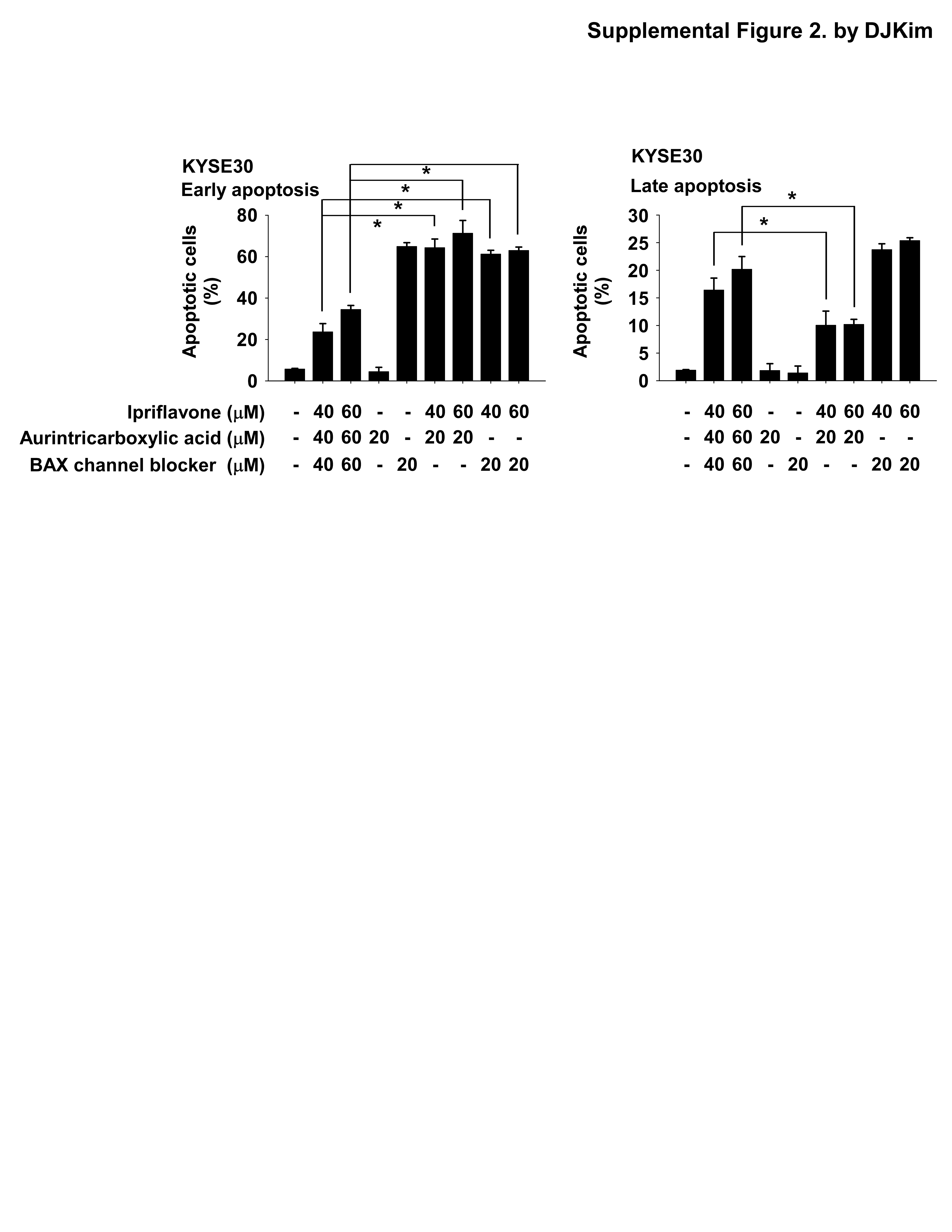

Supplement: Supplementary file 2 [file Image_2.jpeg]

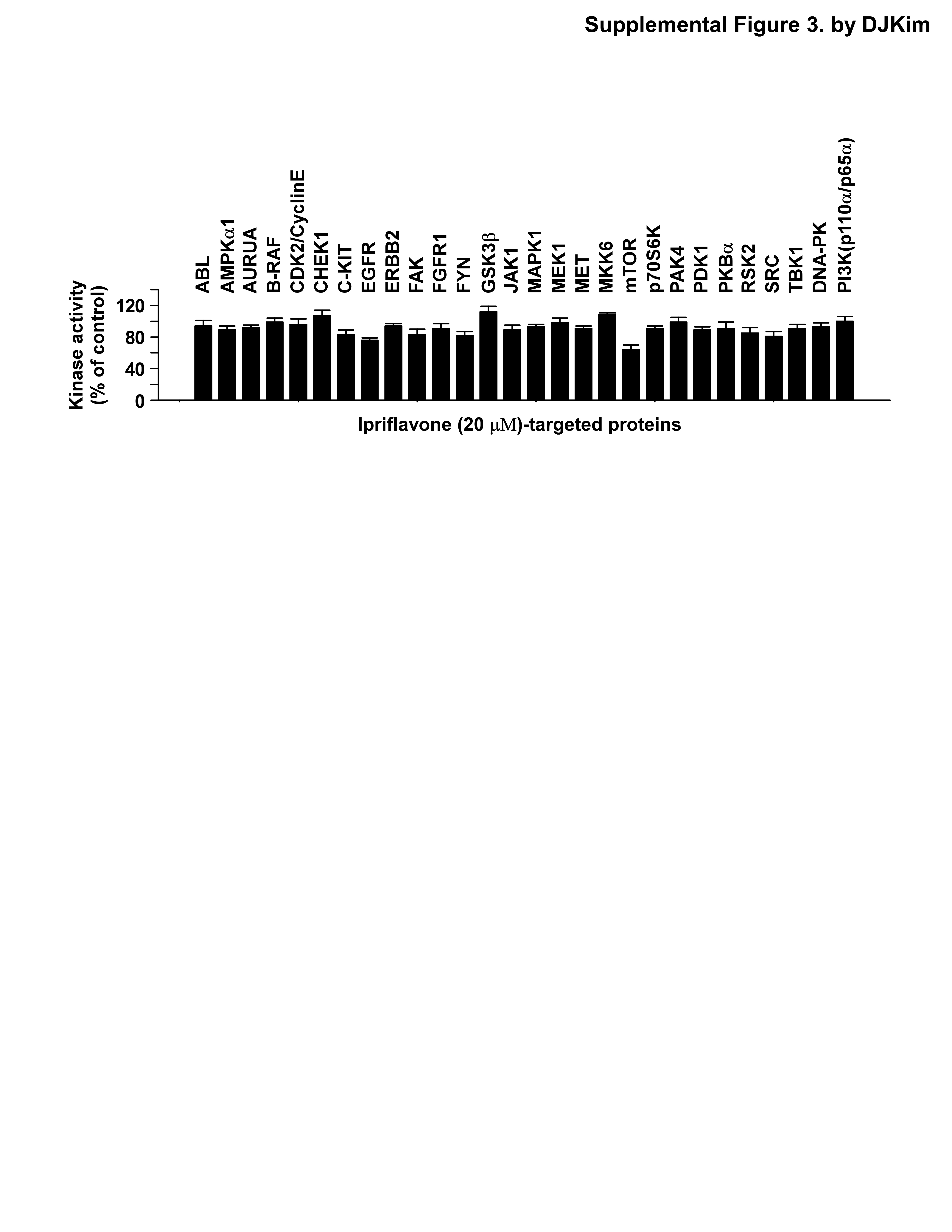

Supplement: Supplementary file 3 [file Image_3.jpeg]

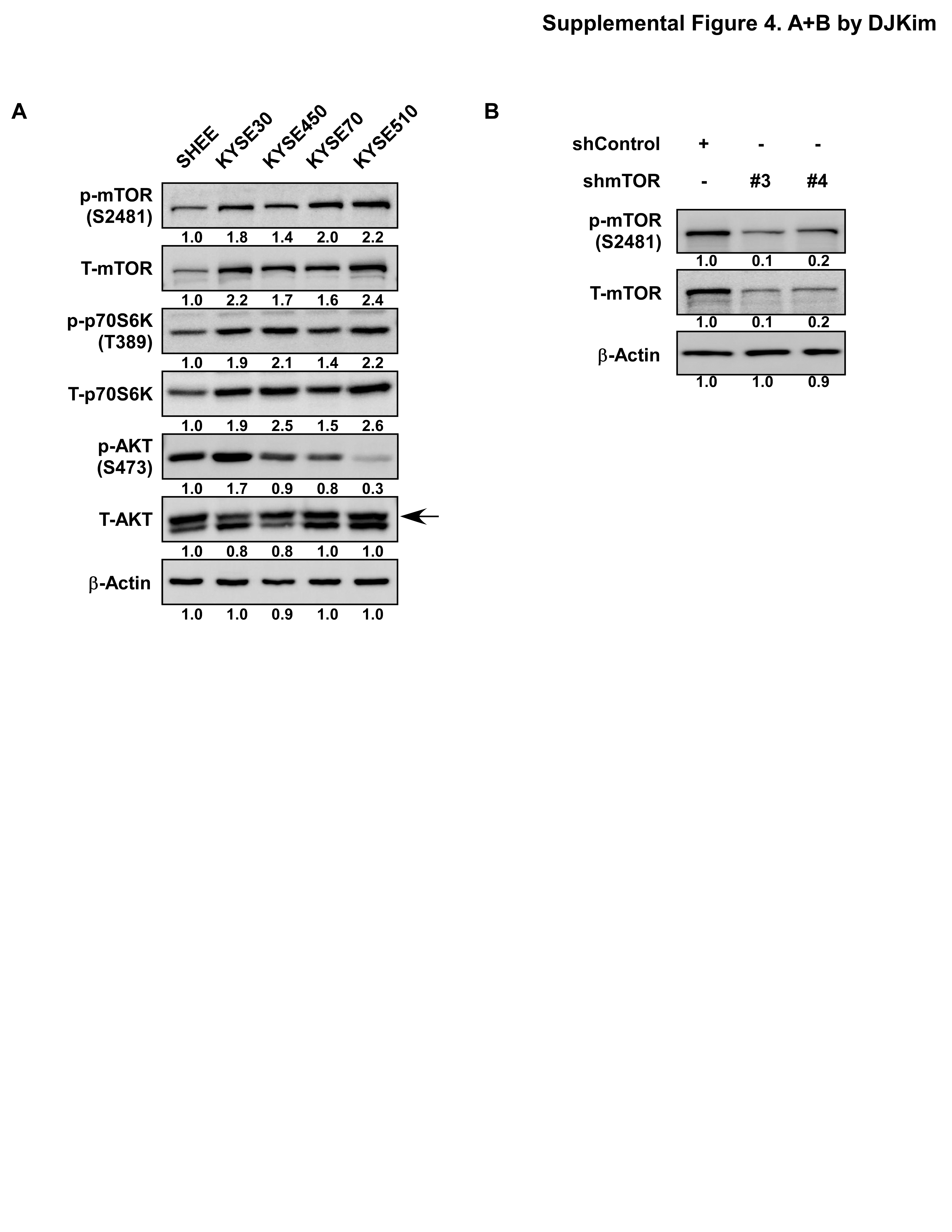

Supplement: Supplementary file 4 [file Image_4.jpeg]

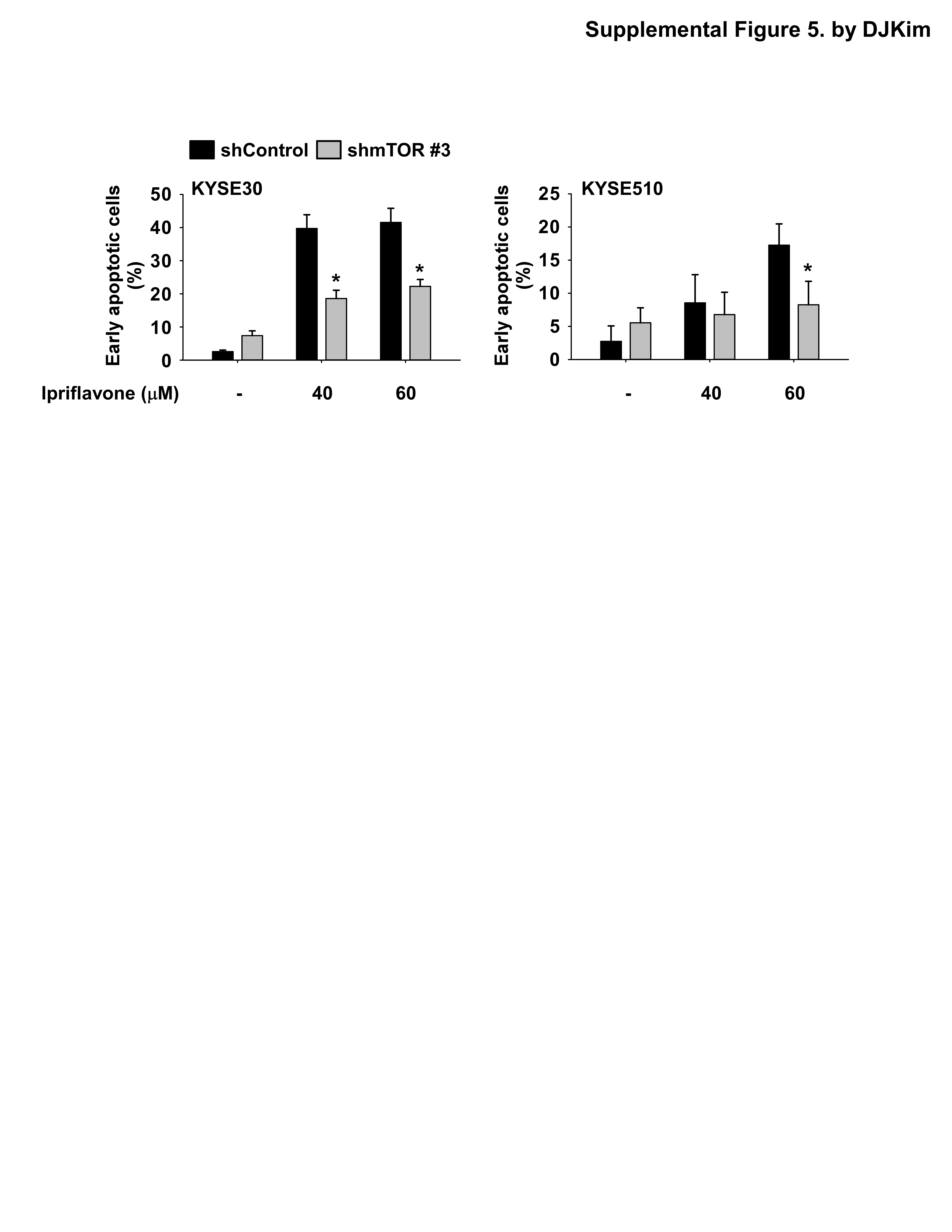

Supplement: Supplementary file 5 [file Image_5.jpeg]

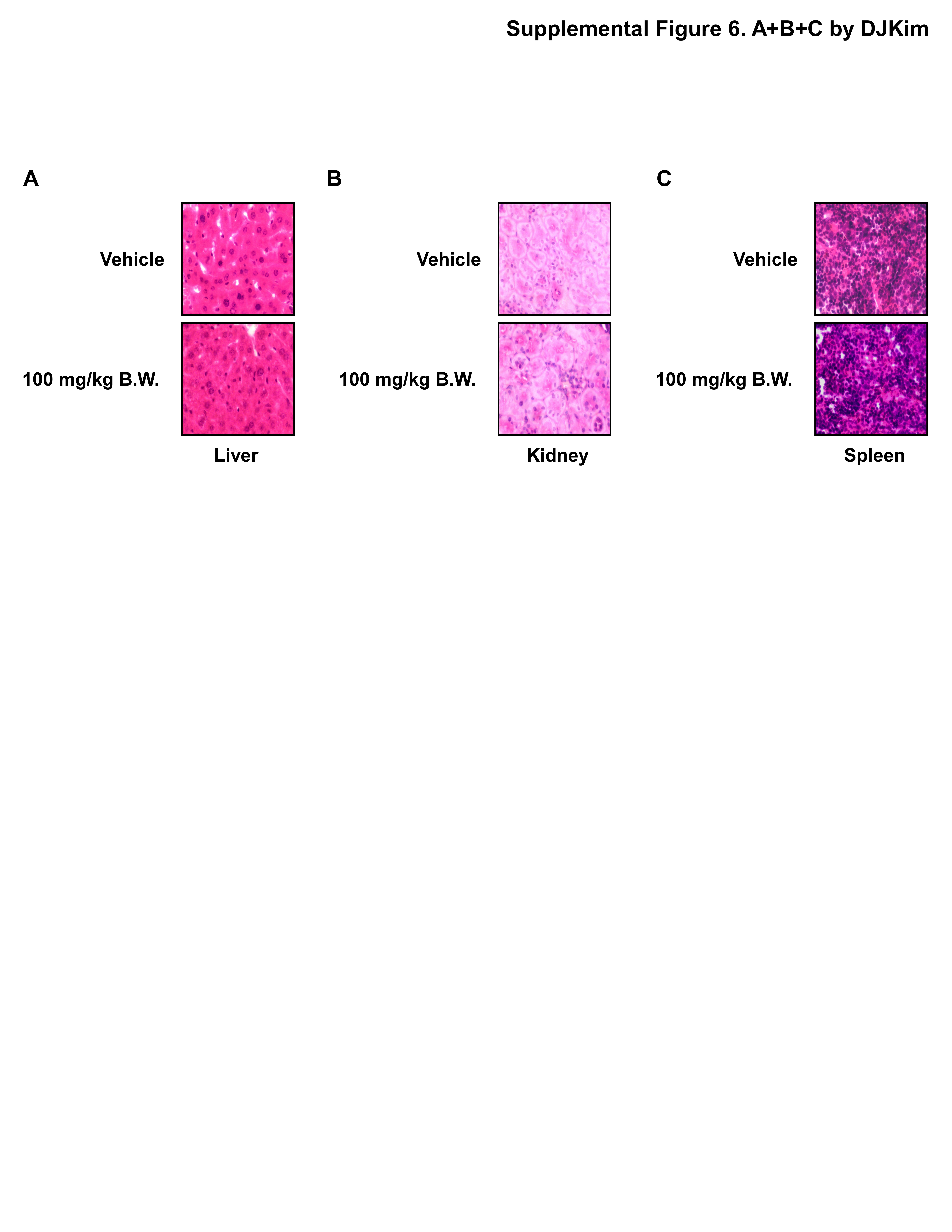

Supplement: Supplementary file 6 [file Image_6.jpeg]

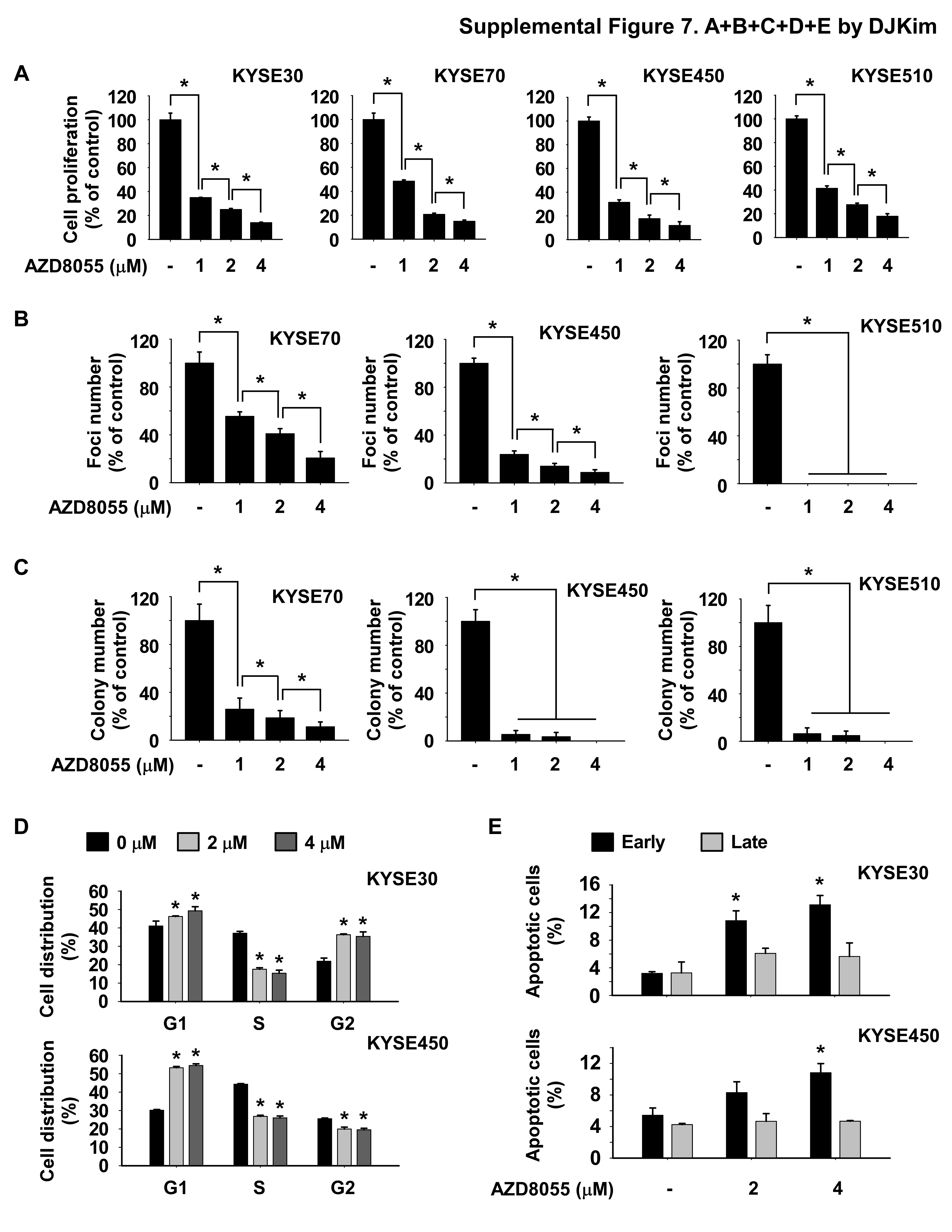

Supplement: Supplementary file 7 [file Image_7.jpeg]
